# A Re-evaluation of Candidate Gene Studies for Well-Being in Light of Genome-Wide Evidence

**DOI:** 10.1007/s10902-022-00538-x

**Published:** 2022-05-17

**Authors:** Margot P. van de Weijer, Dirk H. M. Pelt, Lianne P. de Vries, Bart M. L. Baselmans, Meike Bartels

**Affiliations:** 1 Department of Biological Psychology, Vrije Universiteit Amsterdam, Amsterdam, The Netherlands; 2 Amsterdam Public Health Research Institute, Amsterdam University Medical Centres, Amsterdam, The Netherlands; 3 Biomedical Technology, Faculty of Technology, Amsterdam University of Applied Sciences, Amsterdam, The Netherlands

**Keywords:** Happiness, Well-being, Candidate gene, Genome-wide association, Gene-environment interaction

## Abstract

Ever since twin-family studies found that a substantial amount (± 40%) of the variation in well-being can be explained by genetic variation, several candidate genes have been proposed explaining this variation. However, these candidate gene and candidate gene-by-environment interaction studies have been surrounded by controversy regarding the validity and replication of their results. In the present study, we review the existing candidate gene literature for well-being. First, we perform a systematic literature search that results in the inclusion of 41 studies. After describing the results of the included studies, we evaluated the included candidate polymorphisms by (1) looking up the results for the studied candidate SNPs in a large well-being genome-wide association study, (2) performing association analyses in UK biobank (UKB) data for the candidate variable number tandem repeats (VNTR) and the APOE ε4 allele, and (3) studying possible candidate interactions with positive and negative environmental moderators using UKB data. We find no support for any of the candidate genes or candidate gene-environment interactions for well-being, with the exception of two SNPs that were chosen based on genome-wide evidence. While the generalizability of our findings is limited by our phenotype and environment definitions, we strongly advise well-being researchers to abandon the candidate gene approach in the field of well-being and move toward genome-wide approaches.

**Supplementary Information:**

The online version contains supplementary material available at 10.1007/s10902-022-00538-x.

## Introduction

Ever since it was discovered that well-being or happiness is a heritable trait (Bartels, 2015; Hamer, 1996; Nes & Røysamb, 2015), well-being researchers have aspired to find the genetic variants responsible for variation in well-being. In 1996, Lykken and Tellegen reported on the first twin analyses and estimated that 44 to 52% of the variance in well-being is associated with genetic variation (Lykken & Tellegen, 2016). In the same year, Hamer predicted that about 10–20 genomic loci would be involved in explaining the heritability of happiness and he proposed a strategy for finding 'happiness genes' by association analysis using loci chosen on the basis of function (Hamer, 1996). Based on earlier biological findings, this quest started with a focus on these so-called *candidate genes* that were hypothesized to hold some sort of biological function important for the biological correlates of well-being. With this in mind several candidate genes for well-being have been investigated.

To illustrate, a polymorphism that was deemed a candidate gene for influencing well-being was the 5-HTTLPR-Variable Number Tandem Repeat (VNTR, a pattern of one or more nucleotides that is repeated, with the number of repeats varying across individuals). The VNTR is located in the promotor region of the *SLC6A4* gene that codes for serotonin (5-HT) transporters, a neurotransmitter commonly implicated in mood disorders and emotional processing (Cowen & Browning, 2015; Lin et al., 2014). The 5-HTTLPR polymorphism was first studied in relation to well-being in a sample of 2574 Americans (De Neve, 2011). It was found that individuals with the short version of 5-HTTLPR reported higher levels of life satisfaction than individuals with the longer version, a finding that failed replication in a study a year later (De Neve et al., 2012). Since then, several studies have examined the association between the 5-HTTLPR VNTR and well-being, producing mixed results (e.g. Gartner et al., 2018; Gohier et al., 2014; Hankin et al., 2011; Hartmann et al., 2014; Matsunaga et al., 2013; Roekel et al., 2016).

While the rationale behind most candidate genes seems reasonable, a large problem of the candidate gene literature in general is that results are mixed and do not seem to replicate (Border, Johnson, et al., 2019; Border, Smolen, et al., 2019). One of the proposed reasons for the lack of replication is that, in retrospect, effect sizes of individual genetic variants are very small (Zhang et al., 2018). Therefore, the samples used in the candidate gene studies in general are too small (ranging from less than a hundred to a couple of thousand individuals), leading to many false positive findings (Border et al., 2019; Border, Smolen, et al., 2019; Dick et al., 2015; Duncan et al., 2019; Hewitt, 2012). Another reason for why candidate gene studies like these were producing mixed effects is that the effects of these genes might depend on the environment. As a result, candidate gene-environment interaction studies started examining the interactions between the genetic polymorphisms and environmental exposures on well-being (i.e., gene-environment interaction). Many of these hypothesized interactions are based on the “*differential susceptibility hypothesis*”. This hypothesis states that individuals who are most vulnerable to adversity/negative environments are also most likely to benefit from supportive/positive environments (Belsky, 2016). Candidate gene-environment interaction studies thus examine if carriers of one or two alleles of a particular gene are more adversely affected by negative environments, or more positively affected by positive environments, than non-carriers. For example, Sheffer-Matan and colleagues found that only individuals with the 5-HTTLPR short allele(s) were happier when they perceived higher social support from their friends (Sheffer-Matan et al., 2019).

Most of the studies mentioned above focus on a definition of well-being or happiness that is most in line with a person’s subjective evaluation of their life and well-being, also referred to as *subjective* well-being. (SWB). Another major well-being definition and line of research is *psychological well-being* (PWB). One of the most influential theories in this respect is Ryff’s theory on PWB, which states that PWB is comprised of the six dimensions: autonomy, environmental mastery, personal growth, positive relationships, purpose in life, and self-acceptance (Ryff, 1989). Importantly, many different well-being definitions exist that focus on SWB, PWB, or a combination of both. While it is beyond the scope of this study to provide an extensive overview of these theories, many well formulated reviews exist, see for example: Lambert et al., 2015, Ryan & Deci, 2001, and Magyar & Keyes, 2019. With respect to genetic studies on well-being, the focus has predominantly been on a subjective well-being definition, since genetic studies for wellbeing leverage very large available samples with DNA information that often have not directly been designed for well-being research but contain well-being assessments anyway.

To more systematically search for genetic variants for complex traits, the so-called Genome-Wide Association (GWA) study approach was introduced (Visscher & Montgomery, 2009). In a GWA study, several millions of single nucleotide polymorphisms (SNPs) are studied in relation to the outcome measure in a hypothesis-free fashion. Using the GWA design, it was quickly discovered that most behavioral/psychological traits are influenced by hundreds to thousands of genetic variants, with most of them carrying tiny effects (Wray et al., 2018). As a consequence, to be able to detect these small effects, performing reliable GWA studies requires large sample sizes, often ranging from a few hundred-thousand to millions of study participants. In the context of well-being, the first genome-wide *hits* were identified in 2016, in a GWA study examining subjective well-being data from almost 300,000 individuals (Okbay et al., 2016). Since then, two more GWA studies have been performed for well-being, both of them examining well-being in the context of a *well-being spectrum* consisting of the highly genetically correlated traits subjective well-being, depressive symptoms, and neuroticism (Baselmans et al., 2019; Turley et al., 2018). By jointly analyzing these traits, Turley and colleagues (Turley et al., 2018) identified 49 genetic variants associated with subjective well-being (*N* = 354,462). Baselmans and colleagues also jointly analyzed these traits in a multivariate fashion resulting in 304 hits, and additionally generated trait-specific estimates for each SNP, and identified 148 and 191 significant hits for life satisfaction and positive affect, respectively (N_obs_ = 2,370,390) (Baselmans et al., 2019). These results reflect a linear positive relation between sample size and the number of hits identified, an effect which has also been demonstrated empirically (Canela-Xandri et al., 2018).

In light of the results that emerged from GWA studies, several researchers started to re-evaluate previous evidence from candidate gene and candidate gene-interaction studies for different traits. In this way, it was found that data from a large population-based sample did not support previous major candidate genes for depression (Border, Johnson, et al., 2019; Border, Smolen, et al., 2019). This includes the 5-HTTPLR gene, studied > 500 times as a candidate gene for depression. Similarly, in a study examining historical candidate genes for schizophrenia in light of results from a large genomic study, no robust evidence was found for the role of the proposed candidate genes (Farrell et al., 2015). Like the aforementioned studies for depression and schizophrenia, the GWAS findings for well-being allow for a re-evaluation of the role of candidate genes for well-being. For the present study, we scan the existing literature for candidate gene studies on well-being and summarize the outcomes of these studies. Second, we look up the studied SNPs in the most recent large GWA study for well-being. Lastly, we examine potential associations of four frequently studied VNTRs (*SLC6A3, DRD4, SLC6A4* (a.k.a. 5HTTLPR)*,* and *MAOA)* and the APOE ε4 allele, with well-being in a large sample from the UK Biobank. In line with the differential susceptibility hypothesis, we also examine potential interactions with positive and negative environmental moderators. In this way, we re-evaluate the role of these candidate genes to explain differences in well-being. With this information we aim to inform the field on pursuing or abandoning (relative expensive) candidate-gene based research approaches.

## Methods

### Systematic Literature Search

Articles were retrieved from *PubMed* (http://www.ncbi.nlm.nih.gov/pubmed) and *Web of Science* (http://apps.webofknowledge.com) through a computerized literature search. A literature search was conducted for studies published up to January 28, 2022. The following search terms were used: “well-being” or “wellbeing” or “well being” or “quality of life” or “satisfaction with life” or “life satisfaction” or “happiness” or “positive affect” or “flourishing” or “meaning in life” or “purpose in life” or “Ryff*” or “PERMA” or “eudai*” or “eudem”, and “genes” or “gene” or “genetics” or “polymorphism”. Studies were included if they (1) examined association(s) between some measure of (mental) well-being and one or more candidate genes (not GWA studies), (2) were peer-reviewed, (3) published in English, and (4) examined these associations in a non-patient/non-clinical human population. Importantly, we only included studies that aimed to examine well-being as a phenotype, and not well-being-related phenotypes such as depressive symptoms.

### SNP Look-up

For our SNP look-up, we used summary statistics from Baselmans et al (2019). Details on this genome-wide association meta-analysis (GWAMA) can be found in the original paper. Briefly, this study performed multivariate GWAMA for four genetically highly related traits: positive affect, life satisfaction, neuroticism, and depressive symptoms, collectively referred to as the well-being spectrum (*N* observations = 2,370,390). The study performed univariate meta-analyses for all traits separately, as well as multivariate analyses where the traits were combined, resulting in 304 significant independent hits. For each candidate gene study identified through our systematic literature search (independent of the outcome of that candidate gene study), we looked up the candidate SNPs in the *N*-weighted GWAMA summary statistics for: (1) life satisfaction, (2) positive affect, and (3) the well-being spectrum composite score. We report the p-values of each of these candidate SNPs in the GWAS summary statistics and compare it to the p-values of the original studies.

### UK Biobank (UKB)

We used data from the UKB to test for potential associations between widely studied VNTRs, APOE ε4, and well-being. The UKB is a UK cohort study with genetic and phenotypic data on approximately 500,000 individuals aged between 40 and 69 years old at recruitment (Bycroft et al., 2018). We included a subset of participants with available well-being data. Well-being was approximated using a happiness question: “In general how happy are you?”. This question was answered by 214,357 participants (on four instances) from the initial touchscreen interview (UKB data-field 4526), and by 157,335 participants who completed an online follow-up questionnaire (UKB data-field 20458). If a participant had data available for multiple instances, we selected the last time-point. Participants could answer the question on scale from 1 to 6 ranging from Extremely happy (1) to Extremely unhappy (6). We reverse-coded the item so that a higher score on the scale reflected a higher level of happiness. To limit bias due to population stratification, we reduced our sample to individuals of Caucasian British ancestry (based on self-report, UKB data-field 22006). In total, this led to a sample size of 226,842 individuals with happiness data.

### VNTR Association Analyses

VNTR data are available in UKB for four highly studied candidate VNTRs in psychiatric genetics, located in *SLC6A3, DRD4, SLC6A4* (*5HTTLPR*)*,* and *MAOA*. Additionally, the moderating SNP rs25531 in *SLC6A4* was imputed to the UKB and included in the present study. These VNTRs (and modifying SNP) were imputed previously in the UKB sample using the Family Transitions Project (FTP), the Center for Antisocial Drug Dependence (CADD), and the Genetics of Antisocial Drug Dependence (GADD) studies as reference panels and show good imputation accuracy (> 0.96 for all four VNTR variants)(Border, Johnson, et al., 2019; Border, Smolen, et al., 2019). *SLC6A3, DRD4,* and *SLC6A4* were imputed as bi-allelic short/long alleles, while the *MAOA* was imputed as bi-allelic risk/wild-type. It is the largest sample for which these VNTRs are available, and the data have been used to study potential associations between depression and these candidate VNTRs (Border et al., 2019; Border, Smolen, et al., 2019). We analyzed additive associations between happiness and the four VNTRs imputed to UKB using linear association analysis in plink (Purcell et al., 2007). Age, sex, genotyping batch, and the first 25 ancestry-informative principal components (PCs) were included as covariates. Since we repeated the analysis six times, once for each VNTR, once for the moderating SNP, and once for APOE, we employed a Bonferroni corrected significance threshold of α = 0.05/6 = 0.008.

### APOE ε4

UKB data was used to test whether the presence of the *APOE ε4* allele was associated with happiness. SNP data for rs429358 and rs7412 was used to determine *APOE* genotypes (*APOE* ε4 present/not present). We tested for association using linear regression models in R, including age, sex, genotyping batch, and the first 25 ancestry-informative principal components (PCs) as covariates.

### Interaction Studies

A subset of the articles identified in our systematic literature search examined gene-environment interactions within the differential susceptibility framework (Bradley et al., 2013; Gartner et al., 2018; Hankin et al., 2011; Kuepper et al., 2012; Martin, et al., 2014; Sheffer-Matan et al., 2019; Sicorello et al., 2020). These studies were performed for *APOE ε4*, the *MAOA* VNTR, *OXTR*, and the *5-HTTLPR* gene (see results and Online Resource Table 1). We tested for interaction with both positive and negative environmental moderators for the VNTRs and *APOE* genes using UKB data. In line with Border and colleagues (Border, Johnson, et al., 2019; Border, Smolen, et al., 2019), we included childhood trauma, adult trauma, and recent trauma as negative environmental moderators. As positive moderators we included frequency of friends/family visits, and ableness to confide. Details on these variables can be found in Online Resource Table 2.

**Table 1:**
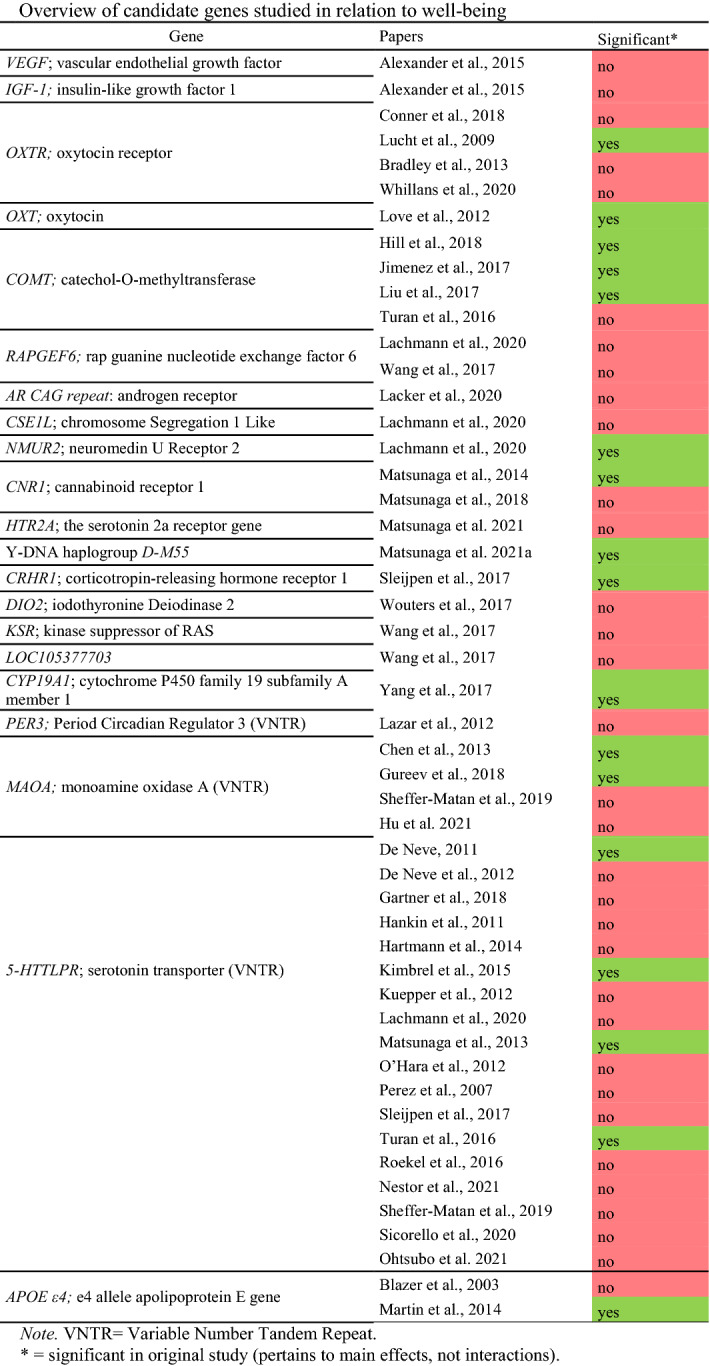
Overview of candidate genes studied in relation to well-being

**Table 2:** P-values of candidate polymorphisms in GWAS

Gene; polymorphism	Papers	paper p-value	PA GWAS p-value	LS GWAS p-value	3-WBS GWAS p-value
*VEGF*; rs699947	Alexander et al. (2015)	.30	.93	.23	.38
*VEGF*; rs833068		.45	.87	.69	.53
*VEGF*; rs3024994		.18	.65	.27	.40
*VEGF*; rs2146323		.49	.45	.50	.58
*VEGF*; rs3025033		.66	.31	.74	–
*VEGF*; rs3025035		.07	.31	.52	–
*IGF-1*; rs2288377		.30	.78	.44	–
*IGF-1*; rs35767		.50	.84	.64	.23
*IGF-1*; rs35765		.93	.63	.98	.40
*IGF-1*; rs7965399		.11	.94	.10	.62
*OXTR;* rs53576	Conner et al. (2018)	.21	.68	.06	.56
	Lucht et al. (2009)—adults	.**045**			
	Lucht et al. (2009)—adolescents	.99			
	Bradley et al. (2013)	n.s			
	Whillans et al. (2020)	.80			
*OXTR*; rs2254298	Lucht et al. (2009) – adults	.07	.04	.70	.27
	Lucht et al. (2009)—adolescents	.44			
	Whillans et al. (2020)	.98			
*OXTR;* rs2228485	Lucht et al. (2009) – adults	.35	.03	.39	.26
	Lucht et al. (2009) – adolescents	.57			
*OXTR*; rs2268498	Whillans et al. 2020	.58	.36	.01	.34
*OXT*; rs4813625	Love et al. (2012)*	**.02**	.28	.46	-
*COMT*; Val158Met (rs4680)	Hill et al. (2018)	**.01**	.33	.84	.91
	Jimenez et al. (2017)	.**01**			
	Liu et al. (2017)	**.02**			
*RAPGEF6*; rs3756290	Lachmann et al. (2020)	.64	.0002	.04	.10
	Wang et al. 2017	.82			
*CSE1L*; rs2075677	Lachmann et al. (2020)	.58	**2.28 × 10**^**–13**^	.002	**2.54 × 10**^**–9**^
*NMUR2*; rs4958581	Lachmann et al. (2020)	**.01**	.0002	.16	–
*CNR1*; rs806377	Matsunaga et al. (2014)	** < .05**	.66	.29	.98
*HTR2A;* r6311	Matsunaga et al. (2021a, 2021b)	.59	–	.60	–
*CRHR1*; rs878886	Sleijpen et al. (2017)	**.004**	.38	.62	–
*DIO2*; Thr92Ala; rs225014	Wouters et al. (2017)	n.s	.43	.52	.81
*KSR2*;rs7973260	Wang et al. (2017)	0.72	.54	.45	8.54 × 10^–5^
*LOC105377703-*rs4481363	Wang et al. (2017)	0.94	.03	.02	**1.28 × 10**^**–13**^
*CYP19A1* Val80; rs700518	Yang et al. (2017)*	** < .001**	.34	.55	.56

*n.s*. non-significant (unreported) p-value. “-” indicates that the SNP was not examined in the relevant GWAMA. P-values indicated in bold are significant according to the original study

^*^ multiple measures of well-being were used, we report the most significant one

Regression analyses where we tested for interactions between *SLC6A3, DRD4, SLC6A4* (5-HTTLPR)*, MAOA* VNTRs, and the rs25531 SNP in *SLC6A4* and our positive and negative moderators were performed in plink. We tested for interactions between our moderators and the presence/absence of the *APOE ε4* allele in R. Happiness, age, and continuous moderators were standardized prior to the analyses. Age, sex, the first 25 ancestry informative PCs, all covariate-by-polymorphism interaction terms, and all covariate-by-moderator interaction terms were included as covariates (Keller, 2014). To test for significance, a Bonferroni corrected significance threshold of α = 0.05/(6 polymorphisms × 3 moderators =)18 = 0.003 was used.

## Results

### Identified Literature

**Fig. 1:**
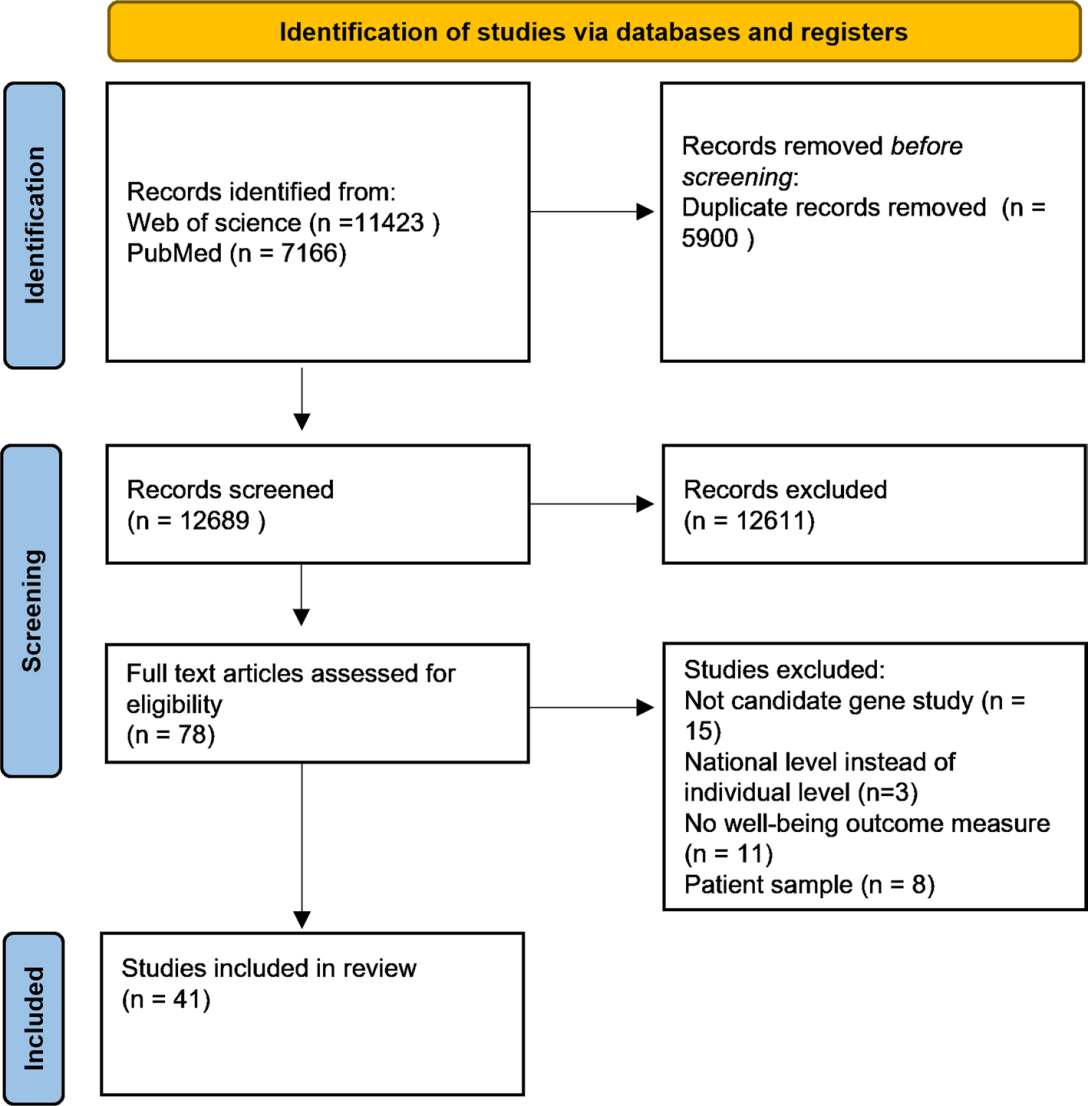
PRISMA flow diagram of the conducted literature search

A PRISMA flow diagram (Page et al., 2021) of our search process is depicted in Fig. 1. Of the 11,400 studies identified in our literature search, 41 were included in the current study. Table 1 provides an overview of these studies and the genetic polymorphisms that were examined. More details on the individual studies can be found in Online Resource Table 3 and 4. Of the 41 included studies, 16 examined the effect of one or more candidate SNPs on a well-being outcome, 1 examined a candidate haplotype polymorphism, 19 examined the effect of a candidate VNTR on a well-being outcome, 3 examined both SNPs and VNTRs, and 2 examined the association between the *APOE ε4* allele and well-being. Some of these studies examined main effects while others also examined interaction effects. The reasons behind studying these genes (and interactions) as candidates in the context of well-being (as stated by the original studies) are listed in Online Resource Table 1.

**Table 3:**
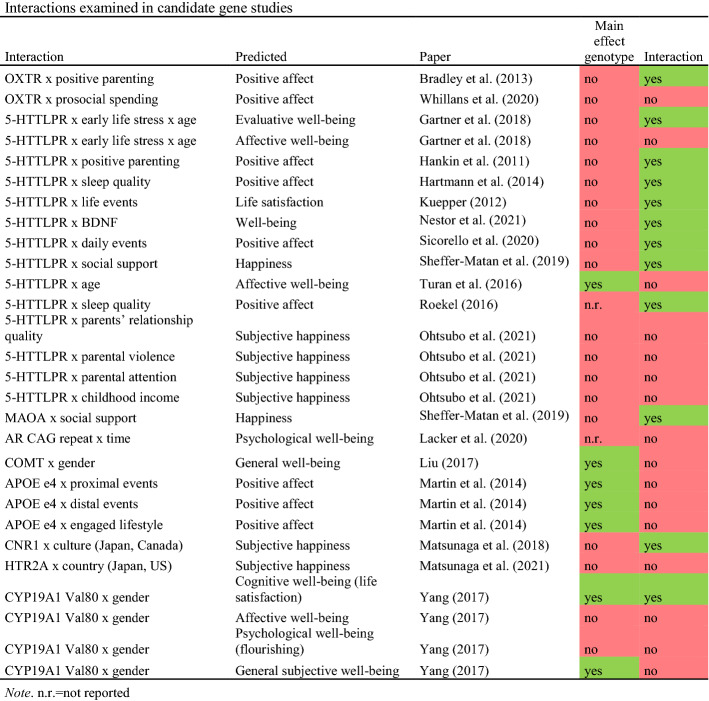
Interactions examined in candidate gene studies

**Table 4:** Results additive association analysis VNTRs UKB

VNTR region	Effect allele	β (SE)	p-value
*DAT1*	Short allele	.002 (.003)	.33
*DRD4*	Long allele	.002 (.003)	.56
*SLC6A4*: rs25531	G	− .002 (.004)	.57
*SLC6A4: 5HTTLPR*	Short allele	− 002 (.002)	.35
*MAOA*	Risk allele	− .003 (.002)	.14

### Summary of Results from Candidate Literature

#### SNPs

An overview of the candidate gene studies that examined associations between well-being and on one or more SNPs is presented in Table 2. Some of these candidate gene studies also included interaction effects, which are discussed in a later section. Candidate gene studies for *VEGF* SNPs, *IGF-1* SNPs, *OXTR* rs2254298, *OXTR* rs2228485, *OXTR* rs2268498, *RAPGEF6* rs3756290, *DIO2* Thr92Ala rs225014, *KSR2* rs7973260, *HTR2A* rs6311, and *LOC105377703* rs4481363 did not find significant associations with well-being. One study found a significant association between *OXTR* rs53576 and well-being in adults (Lucht et al., 2009), but this result was not replicated in adolescents or in other studies. Besides this SNP, six other candidate SNPs were reported to be significantly associated with well-being: *OXT* rs4813625 (Love et al., 2012), *COMT* rs4680 (Hill et al., 2018; Jimenez et al., 2017; Liu et al., 2017), *NMUR2* rs4958581 (Lachmann et al., 2020), *CNR1* rs806377 (Lachmann et al., 2020), *CRHR1* rs878886(Sleijpen et al., 2017), and *CYP19A1* rs700518 (Yang et al., 2017) (without replication efforts). One study that is not mentioned in Table 2 is a study that examined the Y-DNA haplogroup DM55, a genetic polymorphism unique to Japan. This study (Matsunaga et al., 2021a) found an association between subjective happiness and DM55, where mean happiness was higher in females than in non-carrier males, but the differences between females and carrier males, and between carrier and non-carrier males were not significant. Since this haplotype is unique to Japan, we were unable to evaluate this study in light of the GWAS results or UKB dataset.

#### VNTRs

Across the candidate gene literature identified through our systematic literature search, 3 VNTRs were studied in relation to well-being: the *MAOA* VNTR, the *5-HTTLPR* VNTR and the *PER3* VNTR (see Table 1). For 5-HTTLPR, 4 studies found statistically significant associations with well-being, while 13 studies did not find significant associations (see Table 1). Four studies examined the relation between well-being and the VNTR region in the *MAOA* gene. Gureev and colleagues (Gureev et al., 2018) found an association between this VNTR and subjective well-being in men, while Chen and colleagues (Chen et al., 2013) found an association between happiness and the *MAOA* VNTR in women, but not men. Sheffer-Matan and colleagues (Sheffer-Matan et al., 2019) did find a significant interaction between *MAOA* and social support from friends, but did not find a significant main effect for *MAOA* on happiness. Lu and colleagues also did not find a significant main effect for MAOA on subjective well-being (Hu et al., 2021). Lastly, Lázár and colleagues (Lazar et al., 2012) examined if there was an association between a VNTR in the PERIOD3 (*PER3*) region and (positive and negative) affect, but did not find a significant effect of genotype on affect.

#### APOE ε4

Two studies examined associations between the *APOE ε4* allele and well-being. Blazer and colleagues (Blazer et al., 2003) examined associations between the ε4 allele and five parameters of quality of life (including a measure of mental quality of life, measured based on a combination of life satisfaction and depression items) in individuals with good quality of life, but did not find any significant association. Martin and colleagues (Martin, et al., 2014) examined whether centenarians carrying the *APOE ε4* allele scored lower on positive affect than centenarians without the *APOE ε4* allele. They found that carriers scored significantly higher on positive affect than non-carriers.

#### Interaction Studies

Eighteen of the included studies examined interaction effects with candidates genes. Details on the interaction studies can be found in Table 3. Across these 18 studies, 28 interactions were studied for 9 candidate genes: *OXTR* (2 studies), *5-HTTLPR* (10 studies), *MAOA* (1 study), *AR* (1 study), *COMT* (1 study), *APOE ε4* (1 study), *CNR1* (1 study), *HTR2A* (1 study), and *CYP19A1* (1 study).

Twelve of the 28 studied interactions were statistically significant. Eight interactions with *5-HTTLPR* significantly predicted various measures of well-being: two-way interactions with positive parenting (Hankin et al., 2011), sleep quality (Hartmann et al., 2014), life events (Kuepper et al., 2012), *BDNF* (Nestor et al., 2021), daily events (Sicorello et al., 2020), social support (Sheffer-Matan et al., 2019), sleep quality (Roekel et al., 2016), and a three-way interaction with early life stress and age (Gartner et al., 2018). One significant interaction was found for the *COMT* gene: an interaction with age (Turan et al., 2016). The remaining three interactions were an interaction between the *MAOA* gene and social support (Sheffer-Matan et al., 2019), an interaction between *CNR1* and culture (Matsunaga et al., 2018), and an interaction between *CYP19A1* and gender (Yang et al., 2017). Only for the latter interaction (between *CYP19A1* and gender to predict cognitive well-being), both a significant main effect for genotype and a significant interaction effect was found (Yang et al., 2017).

### Evaluation of Results from Candidate Literature

#### SNP Look-up

**Fig. 2:**
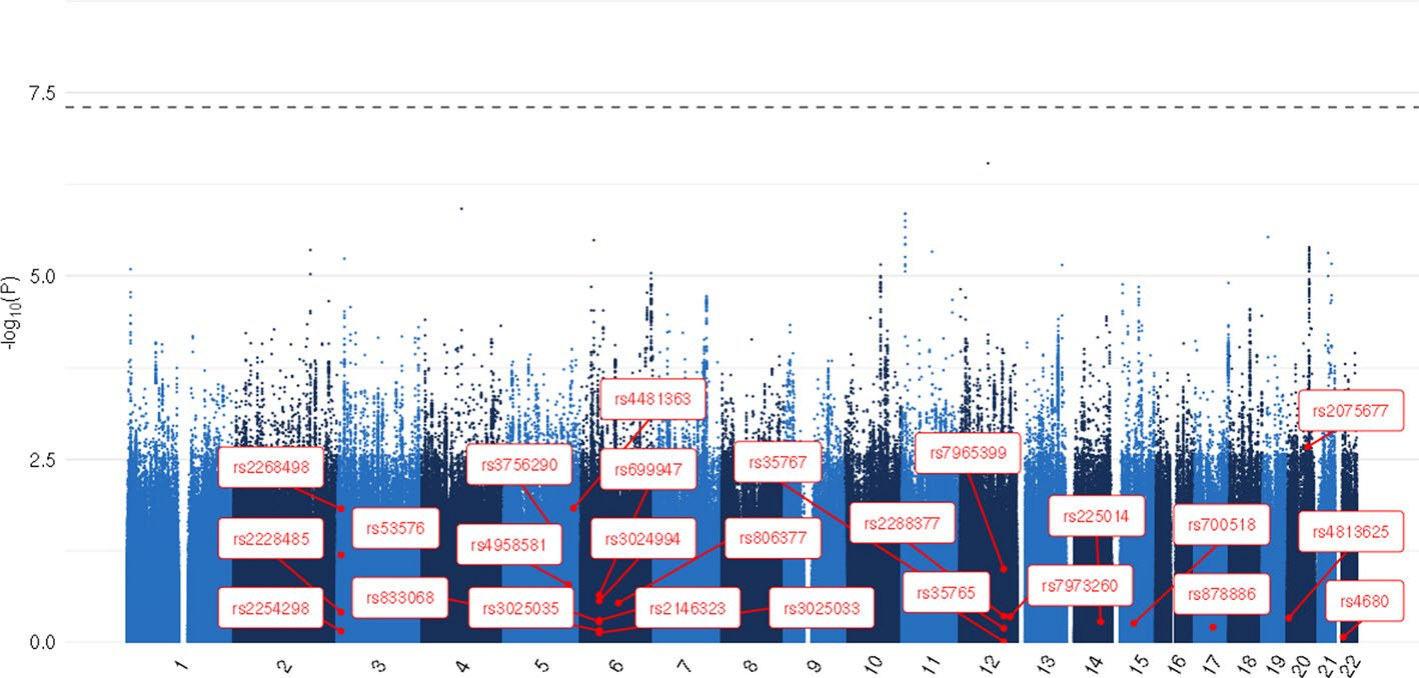
Life satisfaction GWAMA Manhattan plot with highlighted candidate SNPs. The dotted line represents the Bonferroni corrected significance threshold

**Fig. 3:**
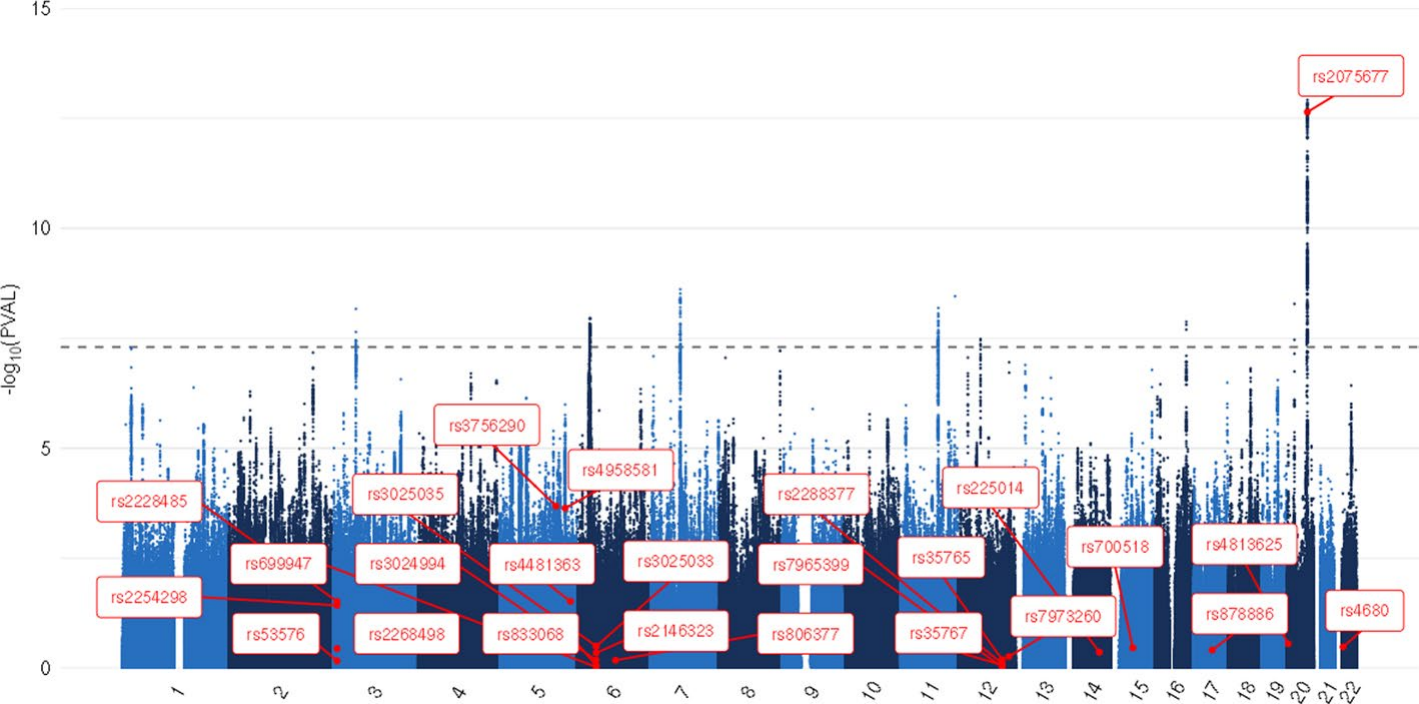
Positive affect GWAMA Manhattan plot with highlighted candidate SNPs. The dotted line represents the Bonferroni corrected significance threshold

**Fig. 4:**
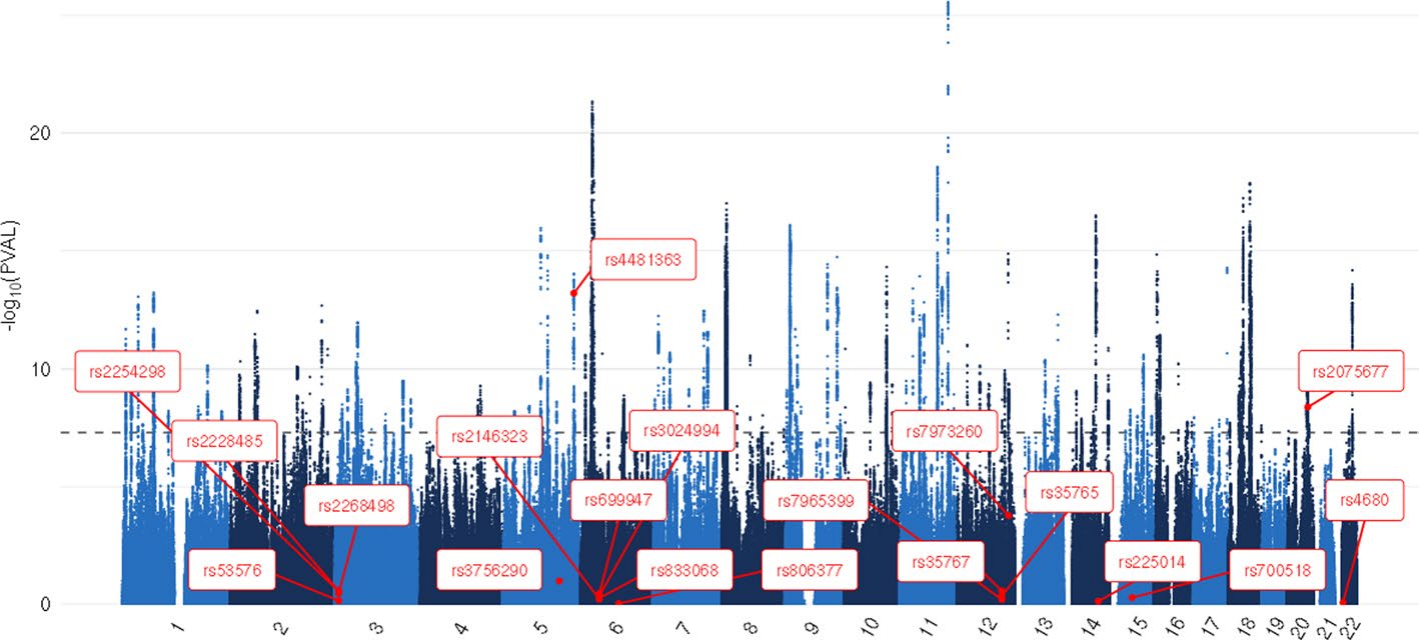
Well-being spectrum GWAMA Manhattan plot with highlighted candidate SNPs. The dotted line represents the Bonferroni corrected significance threshold

For all candidate gene studies identified through our literature search that examined individual SNPs, we looked up the relevant SNPs in summary statistics from the GWA meta-analyses for life satisfaction, positive affect, and the well-being spectrum from Baselmans and colleagues (Baselmans et al., 2019). Table 2 lists these SNPs, the p-values in the original studies (rounded to 2 decimals), and the p-values in these GWA studies. When a “-” is presented instead of a p-value, it means the relevant SNP was not present in the GWAS summary statistics. Figures 2, 3, 4 depict Manhattan plots for life satisfaction, positive affect, and the well-being spectrum with the candidate SNPs highlighted. Two SNPs were significant at a genome-wide level (*p* = 5 × 10^–8^): *CSE1L*- rs2075677 & *LOC105377703*-rs4481363. Importantly, in the candidate gene study where these SNPs were examined (Lachmann et al., 2020), the SNPs were selected based on evidence from an earlier genome-wide association study (Okbay et al., 2016). None of the other SNPs, and thus candidate genes, were significantly associated with life satisfaction, positive affect or the well-being spectrum composite score.

#### VNTR Association Analyses UKB

Using data from UKB, we analyzed if there was an association between happiness and four commonly studied VNTRs (including the *MAOA* and *5-HTTLPR* VNTR), and a moderating SNP in the *5-HTTLPR* region. Results from our association analysis can be found in Table 4. None of the VNTRs or the moderating SNP were significantly associated with happiness (all *p* > 0.008).

#### APOE ε4 Association Analysis

In the present study, the *APOE* genotype distribution (ε2/ε2: 0.6%, ε2/ε3: 12.4%, ε3/ε3: 58.5%, ε2/ε4: 2.5%, ε3/ε4: 23.6%, and ε4/ε4: 2.3%) was comparable to that of other studies (Blazer et al., 2003; Kuo et al., 2020). There was no mean difference in happiness between individuals with the *APOE ε4* allele (*M* = 4.53, *SD* = 0.75), and individuals without the *APOE* ε4 allele (*M* = 4.53, *SD* = 0.76) (*t* = -0.41, *p* = 0.685). We did not find a significant association between *APOE ε4* allele presence and happiness (*β* = 0.0004, *SE* = 0.003, *p* = 0.899).

#### Interaction Analyses UKB

Using UKB data, we tested for interactions of three negative environmental moderators (childhood trauma, adult trauma, and recent trauma) and two positive environmental moderators (frequency of friends/family visits and ableness to confide) with *SLC6A3, DRD4, SLC6A4* (5HTTLPR)*, MAOA* VNTRs, the rs25531 SNP in *SLC6A4*, and the *APOE e4* allele. The results are shown in Table 5. While all of the environmental moderators had a significant main effect on well-being (p-values ranged between 5.48 × 10^–309^ and 2.28 × 10^–15^), none of the polymorphisms or interactions between moderator and polymorphism were significant.

**Table 5:** Results interaction analyses

		Main effect moderator		Main effect polymorphism		Interaction	
Polymorphism	Moderator	β (SE)	p	β (SE)	p	β (SE)	p
*DAT1*	Childhood Trauma	− .316 (.02)	**2.94 × 10**^**–43**^	.001 (.02)	.955	.017 (.01)	.112
	Adult Trauma	− .272 (.02)	**8.76 × 10**^**–42**^	.001 (.02)	.935	.004 (.01)	.672
	Recent Trauma	− .273 (.03)	**5.01 × 10**^**–17**^	.001 (.02)	.960	.014 (.02)	.371
	Family/Friend visits	.064 (.01)	**1.97 × 10**^**–19**^	.005 (.01)	.689	− .003 (.003)	.438
	Able to confide	.261 (.01)	**2.33 × 10**^**–291**^	− .011 (.01)	.301	.001 (.003)	.680
*DRD4*	Childhood Trauma	− .307 (.02)	**1.42 × 10**^**–41**^	.010 (.02)	.537	.001 (.01)	.942
	Adult Trauma	− .266 (.02)	**1.35 × 10**^**–40**^	.013 (.02)	.445	− .011 (.01)	.277
	Recent Trauma	− .261 (.03)	**9.71 × 10**^**–16**^	.004 (.02)	.827	− .015 (.02)	.364
	Family/Friend visits	.060 (.01)	**1.30 × 10**^**–17**^	− .009 (.01)	.464	.005 (.004)	.152
	Able to confide	.259 (.007)	**8.21 × 10**^**–289**^	− .009 (.01)	.444	.007 (.004)	.060
*SLC6A4:*	Childhood Trauma	− .310 (.02)	**6.26 × 10**^**–44**^	.003 (.03)	.900	.024 (.02)	.184
*rs25531*	Adult Trauma	− .272 (.02)	**1.30 × 10**^**–43**^	− .003 (.03)	.911	.009 (.02)	.541
	Recent Trauma	− .267 (.03)	**4.98 × 10**^**–17**^	.008 (.03)	.778	.003 (.03)	.924
	Family/Friend visits	.062 (.007)	**4.98 × 10**^**–19**^	− .004 (.02)	.849	.007 (.006)	.236
	Able to confide	.263 (.007)	**5.84 × 10**^**–309**^	− .011 (.02)	.550	− .007 (.006)	.241
*SLC6A4:*	Childhood Trauma	− .302 (.024)	**3.76 × 10**^**–37**^	.002 (.01)	.866	− .005 (.01)	.594
*5HTTLPR*	Adult Trauma	− .266 (.02)	**1.26 × 10**^**–37**^	.007 (.01)	.645	− .005 (.01)	.552
	Recent Trauma	− .270 (.03)	**2.28 × 10**^**–15**^	.002 (.01)	.875	.003 (.01)	.810
	Family/Friend visits	.065 (.007)	**1.33 × 10**^**–18**^	.006 (.010)	.531	− .003 (.003)	.372
	Able to confide	.263 (.007)	**5.03 × 10**^**–274**^	.005 (.010)	.585	− .001 (.003)	.646
*MAOA*	Childhood Trauma	− .312 (.02)	**3.56 × 10**^**–42**^	− .010 (.01)	.404	.007 (.008)	.390
	Adult Trauma	− .267 (.02)	**1.94 × 10**^**–40**^	− .010 (.01)	.691	− .004 (.007)	.560
	Recent Trauma	− .268 (.03)	**3.81 × 10**^**–16**^	− .011 (.01)	.365	.001 (.01)	.968
	Family/Friend visits	.061 (.007)	**2.09 × 10**^**–17**^	− .004 (.009)	.664	.003 (.003)	.230 lePara>
	Able to confide	.263 (.007)	**1.09 × 10**^**–294**^	− .0004 (.01)	.960	− .002 (.003)	.466
*APOE e4*	Childhood Trauma	− .309 (.02)	** < 2 × 10**^**–16**^	.027 (.02)	.217	.013 (.02)	.385
	Adult Trauma	− .271 (.02)	** < 2 × 10**^**–16**^	.029 (.02)	.201	.002 (.01)	.880
	Recent Trauma	− .267 (.03)	** < 2 × 10**^**–16**^	.029 (.02)	.180	.010 (.02)	.647
	Family/Friend visits	.061 (.007)	** < 2 × 10**^**–16**^	.004 (.02)	.791	.005 (.005)	.267
	Able to confide	.255 (.007)	** < 2 × 10**^**–16**^	.004 (.02)	.791	− .003 (.005)	.457

Values in bold are significant

## Discussion

This study set out by reviewing the candidate gene literature for well-being. To this end, we performed (1) a systematic literature search to identify all the well-being candidate gene literature, (2) a look-up of the studied genomic locations in the largest well-being GWA study, (3) association analyses for commonly studied VNTRs and *APOE* with well-being in UKB data, and (4) association analyses of interactions between negative and positive environmental moderators and the VNTRs and *APOE* in relation to well-being.

In total, 41 studies were included in the present review. Nineteen of these studies examined candidate SNPs in relation to well-being. With sample sizes ranging from less to a hundred to a few thousand, the results from these studies were mixed. Additionally, 20 studies examined potential associations between different VNTRs (*5-HTTLPR*, *MAOA* & *PER3*) and well-being, also producing mixed results. A look up of these SNPs in the GWAS by Baselmans and colleagues (Baselmans et al., 2019) revealed no significant associations with life satisfaction, positive affect, or a 3-trait well-being spectrum, with the exception of 2 SNPs across 2 candidate gene studies. In these 2 candidate studies, these SNPs were not significant, but were selected because they were significant in an earlier GWA study (Okbay et al., 2016). Next, our own association analyses between 5 commonly studied VNTRs (including *5-HTTLPR* & *MAOA*) in over 200,000 individuals of the UKB did not result in significant results. While we were not able to study the association between *PER3* and well-being, this gene was not significantly associated with well-being in the original candidate gene study (Lazar et al., 2012). Similarly, we failed to identify a significant association between the *APOE ε4* allele and well-being in our UKB analyses. Lastly, 18 of the included studies examined the potential effects of interactions between environmental moderators and genetic polymorphisms. Most often, these studies are based on the differential susceptibility hypothesis stating that individuals who are most vulnerable to adversity/negative environments are also most likely to benefit from supportive/positive environments (Belsky, 2016). To this end, we examined interactions between three negative and two positive environmental moderators and the included VNTRs and the *APOE ε4* allele. None of the interactions significantly predicted well-being in the UKB sample.

Taken together, these results indicate that the candidate gene approach is largely unsuitable for studying both main genotypic effects and gene-environment interactions in the context of a polygenic complex trait like well-being. While well-being is a heritable trait and many genetic polymorphisms have been associated with well-being in a genome-wide context, individual genetic effects are extremely small, meaning that extremely large sample sizes are required to detect them. This is even more so the case for interaction effects, which are harder to detect than main effects, increasing the required sample size even further (Aschard et al., 2012). Most candidate gene studies up until now have employed sample sizes too small to detect these effects, ranging from less than 100 to a couple of 1000 individuals (Online Resource Table 3) (average *N* = 774). In a study by Okbay and Rietveld (2015), Bayesian power analyses indicated that in a scenario of an expected effect size of R^2^ = 0.01 (which is much larger than we would expect for a single variant for well-being) and a sample size of *N* = 1000 (and a prior belief in the association of 1%), the power of the test is only 17%. Moreover, the posterior belief in a significant association was still only 3%. Additionally, the genetics and biology of well-being are too complex to easily form hypotheses on potentially relevant genetic polymorphisms, leading to a lack of support for popular hypotheses such as the *5-HTTLPR* hypothesis. We therefore strongly encourage researchers in the well-being field interested in genetic (and gene-environment) effects to abandon the candidate gene approach and to take on the hypothesis-free GWA approach or use the summary statistics for follow-up analyses.

These summary statistics can be used to calculate so called polygenic scores (PGS): quantitative measures that summarize the estimated effect of many genetic variants on an individual's phenotype, typically calculated as a weighted sum of trait-associated alleles. For example, using summary statistics from the same well-being GWAS as used in this study, Jamshidi and colleagues created PGS to predict different (subjective and psychological) well-being measures (Jamshidi et al., 2020). While they found an indication for differences in predictive power across different measurement instruments, none of these differences were statistically significant. Moreover, Patel and colleagues used a PGS for well-being, based on GWAS summary statistics from Turley et al. (Turley et al., 2018) to study the association between subjective well-being and self-employment. They found that the genetic predisposition for well-being (in the form of this PGS) is positively associated with the likelihood of self-employment and earnings. By using a genetic instrument to examine the consequences of well-being on self-employment, the study extends existing literature that mainly focused on potential benefits of self-employment for well-being (Patel et al., 2020). Furthermore, the summary statistics of these large GWAS studies can be used to study direction of causation in a Mendelian Randomization framework. For example, using this approach de Vries and colleagues (de Vries et al., 2021) report causal relations from well-being to resilience, and Zhou and colleagues (Zhou et al., 2021) report bidirectional causal associations of insomnia with depressive symptoms and subjective well-being.

Our findings are prone to several limitations. First, we relied on a broad definition of well-being that was not limited to one specific well-being construct. We included candidate gene studies that used various measures of both psychological and subjective well-being. However, almost all included studies used a subjective well-being outcome measure for their analyses. However, effect sizes for psychological well-being (in the form of meaning in life) for the only GWAS on this topic show effect sizes in the same range as for subjective well-being (Baselmans & Bartels, 2018). Therefore, we do not expect large effects for individual genetic variants for psychological well-being, leading to the same complication for candidate gene studies on this definition of well-being.

For our SNP look up, we examined results from the Baselmans et al. GWAS (Baselmans et al., 2019), including results for positive affect (including happiness measures), life satisfaction, and the well-being spectrum. For our VNTR/*APOE* analyses, we used a UKB measure of happiness. Since our own well-being definitions were not always the same as the constructs used in the different candidate gene studies, we assume that the genetic architecture of different well-being constructs is largely similar, which is confirmed in earlier work reporting high genetic correlations between measures of subjective and psychological well-being (Bartels & Boomsma, 2009; Baselmans & Bartels, 2018). Second, while we included different positive and negative environmental moderators in our interaction analyses to test the differential susceptibility hypothesis, they are not identical to the measures used in the included candidate gene-environment studies. It may be the case that we would have found different results if we included different environmental moderators, but given the extremely small effect sizes of significant SNPs, and the abundance of literature showing no evidence for candidate gene-by-environment interactions (Dick et al., 2015; Duncan & Keller, 2011), we believe it is unlikely that strong gene-environment effects can be found for individual SNPs. Additionally, the GWA results were based on individuals from European ancestry and the VNTR/APOE analyses were performed on UK participants. There are currently no large-scale genome-wide studies on the genetics of well-being in non-Caucasian individuals, limiting our ability to draw conclusions on those populations. For two unrelated phenotypes, height and BMI, a substantial genetic correlation was found between European and non-European samples (Guo et al., 2021). While this does not necessarily generalize to well-being, it does give a first indication that a proportion of GWAS findings in Europeans are likely applicable to non-Europeans.

While the generalizability of our findings is limited by our phenotype and environment definitions, the strength of this study is that the analyses were performed in a much larger sample than those of the included candidate gene studies. In order to continue the progress made in the area of well-being genetics, we advise to abandon the candidate gene approach and move toward well-powered genome-wide approaches, in line with conclusions from earlier work reviewing candidate gene studies for other phenotypes (Border, Johnson, et al., 2019; Border, Smolen, et al., 2019; Duncan et al., 2019). In the context of gene-environment research, it is unlikely that any individual SNP or gene will have a strong interaction effect with an environmental moderator. Instead of focusing on specific candidate SNPs or candidate genes in gene-environment research, an alternative is to look at the joint effect of many well-being associated SNPs, for instance in the form of polygenic scores. These scores are based on GWA summary statistics and reflect an individual’s genetic propensity for a trait of interest. In this way, we might be able to investigate whether the effect of environmental factors is different for people with a different genetic susceptibly – measured across the whole genome rather than a single SNP–for well-being. Moving toward these data-driven approaches will allow us to not only learn more about the biology and genetics of well-being, but will also help us to better understand individual differences in both well-being itself and differences in how people are impacted by environmental factors.

## Supplementary Information

Below is the link to the electronic supplementary material.

Supplementary file1 (XLSX 31 kb)
